# Identification and Ranking of Binding Sites from Structural Ensembles: Application to SARS-CoV-2

**DOI:** 10.3390/v16111647

**Published:** 2024-10-22

**Authors:** Maria Lazou, Ayse A. Bekar-Cesaretli, Sandor Vajda, Diane Joseph-McCarthy

**Affiliations:** 1 Department of Biomedical Engineering, Boston University, Boston, MA 02215, USA; mlzs@bu.edu (M.L.); vajda@bu.edu (S.V.); 2 Department of Chemistry, Boston University, Boston, MA 02215, USA; aybike19@bu.edu

**Keywords:** target evaluation, binding site assessment, ligandability, FTMove, hotspot mapping, SARS-CoV-2 drug targets

## Abstract

Target identification and evaluation is a critical step in the drug discovery process. Although time-intensive and complex, the challenge becomes even more acute in the realm of infectious disease, where the rapid emergence of new viruses, the swift mutation of existing targets, and partial effectiveness of approved antivirals can lead to outbreaks of significant public health concern. The COVID-19 pandemic, caused by the SARS-CoV-2 virus, serves as a prime example of this, where despite the allocation of substantial resources, Paxlovid is currently the only effective treatment. In that case, significant effort pre-pandemic had been expended to evaluate the biological target for the closely related SARS-CoV. In this work, we utilize the computational hot spot mapping method, FTMove, to rapidly identify and rank binding sites for a set of nine SARS-CoV-2 drug/potential drug targets. FTMove takes into account protein flexibility by mapping binding site hot spots across an ensemble of structures for a given target. To assess the applicability of the FTMove approach to a wide range of drug targets for viral pathogens, we also carry out a comprehensive review of the known SARS-CoV-2 ligandable sites. The approach is able to identify the vast majority of all known sites and a few additional sites, which may in fact be yet to be discovered as ligandable. Furthermore, a UMAP analysis of the FTMove features for each identified binding site is largely able to separate predicted sites with experimentally known binders from those without known binders. These results demonstrate the utility of FTMove to rapidly identify actionable sites across a range of targets for a given indication. As such, the approach is expected to be particularly useful for assessing target binding sites for any emerging pathogen, as well as for indications in other disease areas, and providing actionable starting points for structure-based drug design efforts.

## 1. Introduction

The identification and evaluation of potential drug targets is a critical step in the drug discovery process. This task, which is both time-intensive and complex, could be significantly streamlined through the application of computer-aided tools that leverage physics-based models and artificial intelligence. The challenge becomes even more acute in the realm of infectious diseases, where the rapid emergence of new viruses, the swift mutation of existing targets [1], and partial effectiveness of approved antivirals [2] can lead to outbreaks of significant public health concern [1,2]. The COVID-19 pandemic, caused by the SARS-CoV-2 virus, serves as a prime example of this. Despite substantial resources being allocated to drug development, the process remains lengthy. Paxlovid is currently the only effective treatment for COVID-19, and its efficacy is only partial, due to COVID-19 rebound in some patients [3]. Notably, the development of Paxlovid was built upon prior research on other coronaviruses, and the biological target as well as a chemical starting point were available prior to the COVID-19 pandemic [4]. This fact highlights the importance of building foundational knowledge in advance of emerging health threats.

A number of research groups have analyzed the role that computational methods, in particular those involving structure-based design, have played in the COVID-19 pandemic response and how their use could be optimized for preventing and mitigating future pandemics [5,6,7,8,9]. Herein, we computationally map binding site hot spots for a set of the nine most promising COVID-19 targets. This work involves assessing each binding site by utilizing FTMove [10] to examine the structural ensembles from the Protein Data Base (PDB), sometimes augmented with ColabFold [11] generated AlphaFold version 2 (AF2) [12] models. Binding site hot spots are areas on the surface of a protein where ligands are expected to bind with the greatest ligand free energy of binding. As such, we also investigate the correspondence of the computationally predicted binding sites hot spots to the comprehensive set of experimentally known, small-molecule binding sites, RNA binding regions and protein–protein interaction (PPI) regions across the set of targets. A dimensionality reduction of the hot spot score descriptors, the FTMove features, by site is performed to separate sites with known binders from those without known binders. In addition, we explore the effect of structure resolution on mapping results. The methods and findings of this research will serve as a foundation for the development of a fully automated platform for the identification of the most promising binding sites on targets of viruses and other pathogens and diseases of interest that could then serve as starting points for a rapid structure-based drug discovery pipeline.

## 2. Materials and Methods

### 2.1. Selection of SARS-CoV-2 Therapeutic Targets

A set of nine SARS-CoV-2 therapeutic/potential therapeutic targets, including both viral and host proteins, was selected for this study. All of the targets included have previously been investigated for COVID-19 treatment to some extent; several are the biological targets for agents that have entered clinical trials for the treatment of COVID-19.

Of the nine targets, four were viral proteins and five were host proteins. The number of structures in the Protein Data Bank (PDB) with more than 90% identity to the FTMove input structure ranged from over 1700 for the Spike Glycoprotein (GP) to seven for TMPRSS2. For eEF1a, eight structures met the identity criteria, but only five were useable, so these were later supplemented with structural models generated using ColabFold [11] (see description below). RdRp, the Janus kinases (JAK1, JAK2, and JAK3), and PLPro have between 40 to 104 structures. Three of the protein targets (Mpro, RdRp, and Spike GP) have FDA approved medications (drugs or vaccines) associated with them. The primary selection criterion for the FTMove input structure was that the structure contained only the target protein and no other proteins. The input RdRp (nsp12) structure (PDB ID 7ED5) had nsp7 and nsp8 bound; however, they were both deleted prior to running FTMove. Details related to each target are summarized in Table 1.

### 2.2. Generation of Structural Model Ensembles for eEF1a

For the protein eEF1a, three of the eight PDB structures matching the identity criteria were structures with significant conformational change from the reference structure (all-atom root-mean-square deviation (RMSD) > 9 Å) As such, we decided to supplement the PDB structures with structural models generated using the ColabFold [11] implementation of the AF2 approach [12]. AF2 is an AI-based structure prediction method that predicts the 3D structure of a protein from its sequence [12]. With self-attention mechanisms as its core, the algorithm begins by learning from an extensive dataset of known protein structures in the PDB. It then performs multi-sequence alignments, comparing the target protein’s sequence with related sequences from other organisms to provide evolutionary context. By predicting distances between pairs of amino acids and the rotational angles, AF2 constructs an initial 3D model. Finally, to improve the accuracy of the model, the algorithm performs a short physics-based energy minimization.

More specifically, structural models were generated for eEF1a using ColabFold [11] executed locally. ColabFold is a reimplementation of AF2 [12] that accelerates the structure prediction process by replacing the sequence similarity search algorithm of AF2 with MMseqs2 (Many-against-Many sequence searching) [13,14]. As with AF2, ColabFold outputs the predicted Local Distance Difference Test (pLDDT) score, which is a per-residue accuracy confidence score for the structural model [12].

The canonical sequence associated with eEF1a in the UniProt database [15] (ID Q05639) was input to ColabFold. ColabFold was run separately with 100 distinct random seeds to generate an ensemble of structural models for the protein. Each run generated five structural models of the protein using templates and Amber [16] minimization and otherwise default parameters, and the model with the highest pLDDT score (highest confidence) was retained. The list of random seeds used are provided in the Supplementary Material.

### 2.3. Identification of Binding Sites Utilizing FTMove

FTMove is a method for binding site identification that incorporates protein flexibility by identifying and ranking binding sites rapidly across an ensemble of structures or structural models for a given protein [10]. It does so by downloading from the PDB all structures with at least 90% sequence identity to the input PDB structure based on a BLAST search [17]. The approximately 300 structures (due to server limitations) with the highest sequence identity to the input PDB are retained. Alternatively, FTMove can be directly provided an ensemble of protein structures/structural models. The structures/structural models are then aligned, and binding site hot spots are calculated for each one using the FTMap program [18]. FTMap identifies hot spots on a protein surface that are expected to contribute most significantly to the binding free energy of a ligand. For each probe type in the set, FTMap utilizes a Fast Fourier Transform (FFT) approach to sample billions of probe positions on rotational and translational grids. The resulting probe positions are scored with a detailed energy expression, which includes van der Waals energy and electrostatic interaction energy, a term describing the hydrophobic contributions of the cavity and a knowledge-based pairwise potential. The 2000 lowest energy poses for each probe type are retained and energy-minimized with CHARMM potential [19]. Minimized probe positions are further clustered, starting with the lowest energy structure. Clusters are then ranked based on their average Boltzmann energies, and the six lowest energy clusters are retained for each of the 16 probe types. Finally, clusters for each probe are clustered into consensus sites, or binding site hot spots that are ranked by the number of probe clusters present. FTMove combines these mapping results into one visualization using the FTSite algorithm [20], which traces a mesh grid around the hot-spot clusters in close proximity over the aligned FTMap results for all of the structures in the set. The final output includes a PyMOL version 3 (Schrodinger, LLC., New York, NY, USA, 2024) format .pse file for displaying the reference structure and the top 15 identified binding sites, along with a table detailing, for each individual structure analyzed, the number of probe clusters at each FTMove binding site. FTMove ranks the binding sites based on a combination of the maximum number of probe clusters, the average number, and the average without zeros across all the structures. FTMap results for each individual PDB structures are also downloadable. For a given structure, a binding site meets the minimal criterion for druggable if it contains 16 or more probe clusters [10].

FTMove has advantages over other binding site identification methods, such as SiteMap [21,22] and PDBspheres [23], in that it analyzes the ensemble of mappings and outputs a composite set of predicted binding sites. Furthermore, since FTMove is based on the FTMap FFT technology, it is able to perform an exhaustive search of the protein surface relatively rapidly. In addition, while SiteMap calculates an energy potential over grid maps at site points, FTMap maps the position of actual small molecular fragments and as such is a close computational analog of experimental fragment-soaking approaches that have provided a substantial basis for fragment-based drug discovery [24,25,26]. Using the FTMove server, the Mpro run, for example, takes approximately 2.3 h to complete, the bulk of which is due to the FTMap calculations. A single FTMap calculation for Mpro (which consists of 306 residues) takes 0.043 min to complete, or a total of 12 CPU minutes over 28 cores (specifically two fourteen-core 2.6 GHz Intel Xeon Gold 6132 processors (Intel, Penang, Malaysia)).

For most of the proteins studied, the PDB ID given in Table 1 was input to FTMove. For JAK2, one structure (PDB ID 5TQ7) with an engineered (not naturally occurring) point mutation [27] was removed. For TMPRSS2, seven PDB structures were downloaded. To increase the structural diversity, we then manually split the dimeric structures (six) by chains. The resulting 13 structure files were then directly submitted to FTMove for mapping. For eEF1a, a ColabFold generated ensemble of 100 structural models was used together with five of the eight PDB structures. Three eliminated structures of eEF1a had undergone a significant conformational change relative to the reference structure as noted above. Unless otherwise stated, FTMove parameters were set to their default values, with “Chain input” specifically set to chain A.

**Figure 1:**
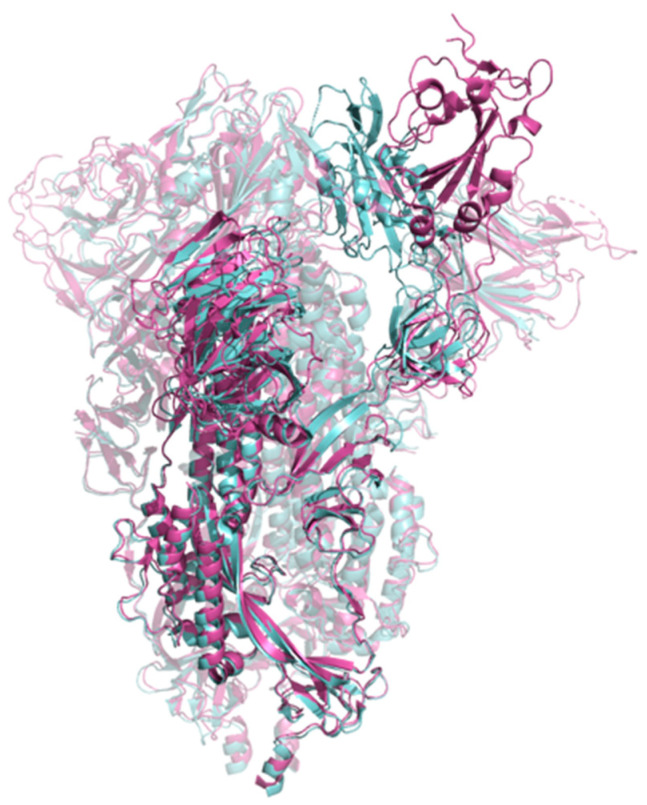
Superposition of SARS-CoV-2 Spike GP with its Receptor Binding Domain (RBD) in the open and closed conformations. A structure with one monomer of the RBD in the open conformation (PDB ID 7DK3) is shown in magenta, and a structure with all three monomers of the RBD in the closed conformation (PDB ID 6VXX) is in cyan.

The SARS-CoV-2 Spike GP exists as a trimer in which each identical monomer can exist in either an open or closed conformation, and the transition between the two conformational states requires a large-scale rigid-body motion [28] (Figure 1). Spike GP in only the open conformation binds to the human angiotensin-converting enzyme 2 (ACE2) receptor as a first step in host cell entry. The ratio of open to closed conformations of the Spike GP monomers in the PDB structures was roughly 3:5. Currently, FTMove cannot process multimer complexes. As such, the input PDB for FTMove was chain A of the pdb code given in Table 1. FTMove then downloads from the PDB all chains with 90% or more identity, so for each trimer structure in the PDB, it separately retrieves three monomer structures.

FTMove analyzed the top 281 Spike GP monomer structures with the greatest sequence identity (100%) to the 6VXX chain A input structure. Of these monomer structures, 92 were in the open conformation, 162 were in the closed conformation, and 27 were eliminated from consideration since they had more than three missing residues in the Receptor Binding Motif (RBM), which is residues 438–506 of the Receptor Binding Domain (RBD) [29]. FTMove was run on the full set, the open set, and the closed set, given the large scale of the conformational change. A list of 254 Spike GP monomer structures used and their conformational state is given in Table S1. The 42 Spike GP RBD only structures were also mapped using FTMove.

### 2.4. Analysis of FTMove Results

For each protein target, FTMove results were analyzed by first visualizing the identified binding sites on the surface of the input structure (or the first input structural model) in PyMOL. For each binding site, in addition to the maximum score (MAX), average score (AVG), and average score without zeros (AVG_WO_0s), the minimum score (MIN), variance (VAR), and percent high score (%HS) as described in Equation (1) were output by FTMove.

(1) $$\%HS=\frac{n}{N}\times 100$$

where n denotes the number of structures with a score of ≥16 and N is the total number of structures analyzed. The threshold of 16 is based on our previous analysis that showed that 16 probe clusters are required for the ability of a hot spot to bind any molecule with nanomolar affinity [17]. In addition, in a postprocessing step, a ratio score was computed that describes %HS relative to that for the strongest site on the protein surface (see Equation (2))

(2) $$Ratio=\frac{{\%HS}_{i}}{{\%HS}_{max}}$$

where *%HS_i_* denotes the %HS of binding site *i*, and *%HS*_max_ is the maximum %HS for the protein overall. Lastly, the number of sites identified by FTMove for the protein was also recorded.

### 2.5. Data Mining to Characterize Binding Sites and Manual Labelling as Ligandable Versus Not

For each protein, FTMove sites 00 to 10, where 00 is the top ranked site, were further characterized based on published data and manually labelled as ligandable vs. not. Specifically, all available PDB structures for the protein (from the same organism as the first input PDB file and with resolution of 4 Å or better) were visualized and aligned with the FTMove sites. In this manual assessment, an FTMove binding site was designated as ligandable if it overlapped with a known binder (small molecule, nucleic acid, or protein–protein interface) in one of the 3D structures (X-ray, cryo-electron microscopy, or NMR). Each ligand-bound site was then categorized based on its functional role, as described in the literature, as active, adjacent, allosteric, protein interface, nucleic acid interaction, or miscellaneous. The miscellaneous designation was applied to sites where a ligand was bound without any known impact on protein function. Conversely, the category “adjacent to active site” was assigned to sites that did not overlap with a ligand in a structure but that were in close proximity (within 5 Å) to a known active site (as defined either by an active site ligand or a catalytic triad residue). This distance threshold is required to account for potential conformational changes upon ligand binding.

### 2.6. Correlation Calculations and Reranking of FTMove Sites

Pearson correlation coefficients between each FTMove feature and the manual ligandability label were calculated to aid in feature selection. In this test, the correlation coefficient varies from 0 to 1, where 1 indicates strong correlation. The results were visualized in heatmaps.

Features with the highest correlation coefficients (>0.55 and >0.45, respectively) were utilized to re-rank the FTMove sites. Specifically, sums of MAX and AVG and of normalized MAX, AVG, %HS, and Ratio were used. These rankings were then compared to the original FTMove ranking based on the sum of MAX, AVG, and AVG_WO_0s for each site. Next, we examined the “recall” for the top five sites after each ranking compared to the “ground truth” ligandability label, where “recall” was the number of true positives divided by the rank of the last true positive.

### 2.7. UMAP Dimensionality Reduction, Sensitivity Analysis, and Final Projection

Each FTMove site was treated as a sample, with the FTMove metrics for that site as the features. Specifically, the FTMove features included were MAX, MIN, AVG, AVG_WO_0s, VAR, %HS, Ratio, and N sites, as described above. Due to the large-scale nature of the conformational dynamics of the Spike GP, only sites from the FTMove calculations on the RBD of the Spike GP were included in the analysis. As such, a total of 81 sites (feature vectors) over nine proteins were included. The Uniform Manifold Approximation (UMAP) [30] was then applied to the data set. UMAP is a dimensionality reduction technique used to visualize high-dimensional data in lower-dimensional space. It aims to preserve the local and global structure of the data, making it effective for revealing intrinsic patterns such as clusters. Thereby, the eight FTMove features were reduced to two UMAP components. All defaults were used in the UMAP generation, apart from the number of nearest neighbors (n_neighbors), which was set to seven to preserve local multidimensional relationships better. N_neighbors is used to construct the neighborhood graph; it determines how many neighboring sample points are considered part of each point’s local neighborhood [30].

Next, the sensitivity of the UMAP components to each feature type was examined. For each feature type, 1000 datasets were generated by randomly shuffling the values for that feature. Then the average Earth Mover’s Distance (EMD) [31] between pairwise distance measures from the original UMAP projection and the shuffled UMAP projection were determined. The larger the EMD, the greater the sensitivity of the UMAP components to that feature.

### 2.8. Analysis of Hot-Spot Score vs. Resolution of Structure

For each Mpro structure (293) included in the FTMove calculation, the resolution and experimental method of determination was extracted from the PDB file. The resolution was plotted versus the score (the maximum number of probe clusters) of each FTMove binding site in each structure, where the color of each data point represented the experimental method of structure determination.

## 3. Results

### 3.1. Comparison of FTMove Sites with Known Sites

**Figure 2:**
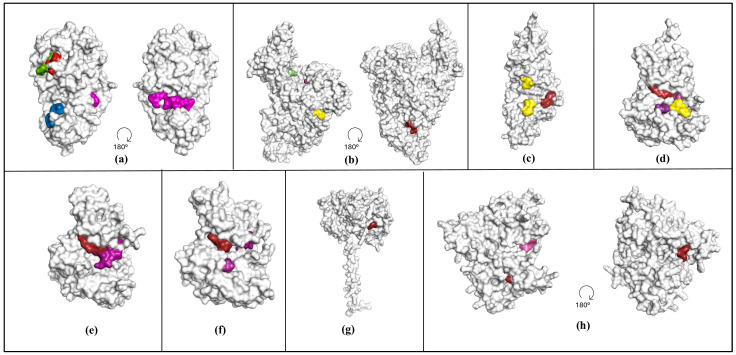
Structures of the eight targets shown in a surface representation, with experimentally known binding sites highlighted by label/category. In (**a**) is shown Mpro (PDB ID: 7S82), (**b**) RdRp (PDB ID: 7ED5), (**c**) PLPro (PDB ID: 6WX4), (**d**) JAK1 (PDB ID: 4EHZ), (**e**) JAK2 (PDB ID: 2XA4), (**f**) JAK3 (PDB ID: 5TTV), (**g**) TMPRSS (PDB ID: 7MEQ), and (**h**) eEF1a (PDB ID: 6ZM0). Red residues correspond to active site, purple to allosteric site, green to adjacent (to active) site, yellow for protein–protein interaction site, pink for nucleic acid binding site, and blue for miscellaneous site.

The main protease, Mpro (also referred to as nsp5 or 3-chymotrypsin-like protease (3CL^pro^)), is a conserved protein among coronaviruses; its main purpose is to cleave polyproteins yielding functional proteins, and as such, it plays a vital role in the replication of the virus [32]. Mpro is the target of the FDA-approved drug Paxlovid (Nirmatrelvir plus Ritonavir) for the treatment of COVID-19 [6]. FTMove identifies six sites manually labeled as ligandable based on the literature review in the top ranked six sites (Figure 2a). Site 00 corresponds to the Nirmatrelvir binding site [6] and is labeled as an active site; Site 02 is adjacent. Site 01/Site 03 and Site 04 correspond to two known allosteric sites, with reported inhibitors and evidence of allosteric effect [33]. Site 05 is a region of compound binding in Mpro that does not affect its functionality [7].

The RNA dependent RNA polymerase, RdRp (nsp12), expresses and replicates the genome of the virus [34]. Together with non-structural protein (nsp) 7 and nsp8 cofactors, it forms a replication transcription complex (RTC), which carries out synthesis, capping, and proofreading of the RNA [34]. RdRp is the target of Remdesivir, a nucleoside inhibitor, FDA-approved drug for the treatment of COVID-19. Remdesivir incorporates itself into a growing RNA strand and halts the synthesis process [35]. FTMove identifies four binding sites labeled as ligandable in the top eight (Figure 2b). Site 01 is at the nsp8 binding site, and Site 02 is at the G-Pocket. The latter site is located on the NiRAN domain of RdRp, which forms a covalent RNA–protein intermediate (RNAylation process) during RNA 5′ capping [36]. The NIRAN domain transfers RNA to GDP, forming a GpppA-RNA (capped RNA) [36]. Yan et al. suggest that compounds such as AT-527 may influence GpppA formation by outcompeting GTP from the G-pocket, therefore stalling the RNA replication process [37]. Site 06 is an RNA binding site and Site 07 is adjacent to the catalytic triad (and adjacent to the Remdesivir binding site). Sites 06 and 07 both have a maximum score less than 16. Site 00 (the top ranked site) does not correspond to any known binding sites on RdRp.

The Papain-Like protease, PLpro, is the largest mature SARS-CoV-2 protein. Its main functions within the viral replication cycle are to cleave polyproteins into functional units for further replication and to remove ubiquitin and interferon-stimulated gene product 15 (ISG15) from ubiquitinated or ISGylated host cell proteins to aid their evasion of the host defensive responses [38]. FTMove identifies four binding sites labeled as ligandable in the top 11 (Figure 2c). Site 02 is the ubiquitin binding site, and Site 04 corresponds to the catalytic site. Site 05 corresponds to the ISG15 binding site, and successful disruption of this protein–protein interaction would be expected to disrupt the functionality of PLpro. Site 10 is a relatively weak predicted site that corresponds to an allosteric site [38].

The Janus kinase (JAK) family represents targets against COVID-19 on the host side, specifically ones that can be inhibited to treat complications of the disease [39]. A number of cytokines signal through the JAK-STAT pathway, including IL-6, IL-2, IL-15, and IL-10, which are elevated in patients with COVID-19 [39]. Three FDA-approved drugs for other indications were repurposed for COVID-19 treatment: Baricitinib, Ruxolitonib, and Tofacitinib, which target JAK1/JAK2, JAK1/JAK2, and JAK1/JAK3, respectively [39]. For JAK1, FTMove identifies four binding sites labeled as ligandable in the top four (Figure 2d). Site 00 is the ATP site; Site 01 is an MT3 allosteric site where Type I, II and III inhibitors bind; Site 02 is the SOCS1 binding site; and Site 03 is an allosteric site at the PDIG motif.

For JAK2, FTMove identifies four binding sites labeled as ligandable in the top five (Figure 2e). Similarly to JAK1, Site 00 and Site 01 correspond to the ATP site and the MT3 allosteric site, respectively. Sites 03 and 04 correspond to an allosteric site near the DFG binding motif.

For JAK3, FTMove identifies three binding sites labeled as ligandable in the top three (Figure 2f). Sites 00, 01, and 02 correspond to the ATP active site, the MT3 allosteric site, and the allosteric site at the PDIG binding motif, respectively.

The host cell protease, TMPRSS2, mediates the second cleavage of the Spike GP, exposing the fusion peptide that governs cell entry of the glycoprotein [40]. FTMove identifies one binding site labeled as ligandable in the top one (Figure 2g). Site 00 corresponds to the location of the catalytic triad site. Inhibitors acting on this site prevent Spike GP maturation and are therefore effective against SARS-CoV-2 [41].

The elongation factor-1 alpha, eEF1a, is a GTPase that escorts aminoacyl-tRNA (aa-tRNA) as an eEF1A(GTP)-aa-tRNA ternary complex to the ribosome [42]. Rapid and accurate mRNA translation requires efficient codon-dependent delivery of the correct aa-tRNA to the ribosomal A site. Conversion of GDP to GTP is essential for regeneration of active eEF1a [43]. Some inhibitors, such as plitidepsin, which binds to the translation inhibition site of eEF1a, have advanced to Phase 2 clinical trials for COVID-19 [44]. The structurally unrelated cyclic peptides didemnin B and ternatin-4 also bind to the eEF1A(GTP)-aa-tRNA ternary complex at the translation inhibition site [45]. FTMove identifies three binding sites labeled as ligandable in the top three (Figure 2h). Site 00 corresponds to the translation inhibition site, Site 01 to the aa-tRNA binding site, and Site 02 to the GDP binding site.

**Figure 3:**
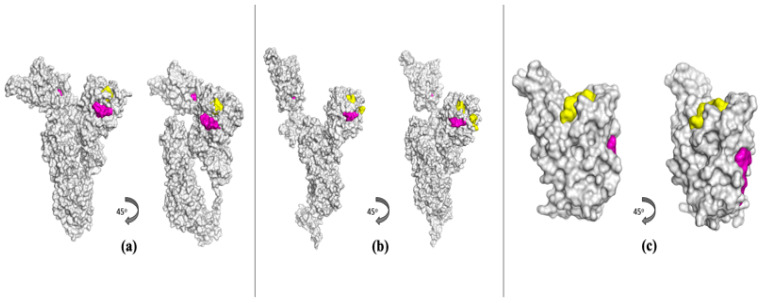
Structures of the Spike GP shown in surface representation with known ligandable binding sites highlighted by label/category. Shown in (**a**) is a Spike GP closed structure (PDB ID: 6ZB5.A) with known binding sites, and in (**b**) is an open structure (PDB ID: 6XM0.B) with known binding sites. In (**c**) is a structure of the Spike GP RBD (PDB ID: 6M0J.B) with known binding sites shown. Red residues correspond to active site, purple to allosteric site, green to adjacent (to active) site, yellow for protein–protein interaction site, pink for nucleic acid binding site, and blue for miscellaneous site.

Spike GP plays a critical role in host cell entry and, as such, has been a target for vaccine as well as therapeutics development. A comparative analysis of the Spike GP structures, illustrated in Figure 3, highlights significant large-scale movement of the Spike GP RBD from the open to closed state; only Spike GP in the open conformation is competent to bind to the human ACE2 receptor [24]. For the ensemble of open and closed structures described in the Section 2 above, FTMove identifies two binding sites labeled as ligandable in the top eight. Site 00 corresponds to the heme binding site, which can allosterically affect the activity of neutralizing antibodies [46]. Site 04 corresponds to the free fatty acid (FFA) binding site; binding to the FFA site has been shown to stabilize Spike in closed conformation, decreasing its interaction with host cells [47]. FTMove was also run on the closed and open conformations separately to see if the results improved. For the set of closed structures, FTMove identifies three binding sites labeled as ligandable in the top seven (Figure 3a). Site 01 is the heme binding site; Site 04 is the FFA binding site; and Site 06 is the N-terminal domain (NTD) antibody binding site. For the open conformation structures, FTMove identifies five sites labeled as ligandable in the top eight. In this case, Site 00 corresponds to the heme binding site, Sites 04, 05, and 06 correspond to the NTD antibody binding site, and Site 07 to the FFA site (Figure 3b). The ACE2 binding site, a protein–protein interaction site, was not observed when mapping the Spike GP overall.

Structures of the Spike GP RBD were also mapped. For the Spike GP RBD, FTMove identifies two binding sites labeled as ligandable in the top four (Figure 3c). Site 01 corresponds to the heme binding site and Site 03 to the ACE2 receptor binding site. Sites 00 and 06 correspond to regions of the Spike GP RBD that would be occupied by other domains of the Spike GP, and as such, they are categorized as PPI sites.

**Figure 4:**
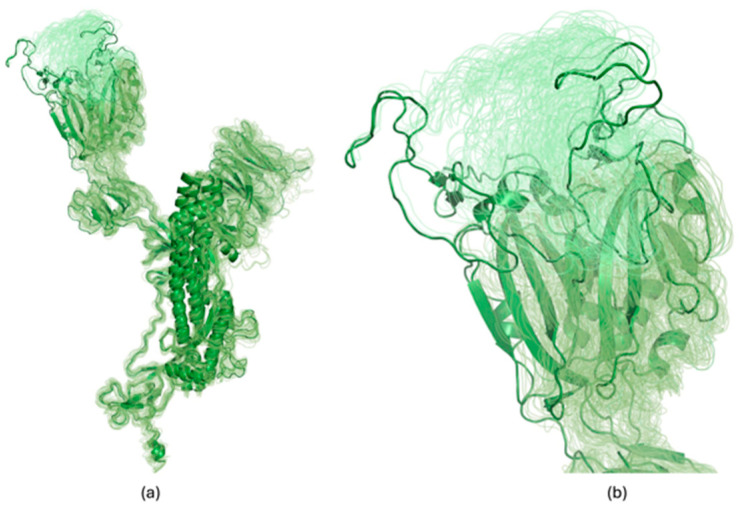
Alignment of open conformations of Spike GP monomers can yield large RMSDs for the Receptor Binding Motif (RBM) region. In (**a**), alignment of the 92 open monomers is shown, with the reference structure and the one with the largest RMSD from that for the RBM highlighted in the ribbon diagram, and (**b**) zooms in on the Receptor Binding Domain (RBD) region. The pairwise alpha-carbon RMSD of the RBM after alignment ranges from 0 to 18 Å. The RBM is the region of the Spike GP RBD that interacts with the ACE2 receptor.

The large-scale conformational change that the Spike GP monomers undergo poses a challenge for the FTSite hot spot clustering step of the FTMove algorithm. FTMove aligns the structures overall, but given the very significant scale of the movement, the ACE2 binding site at the tip of the RBD (Receptor Binding Motif) is the least well aligned (see Figure 4). This lack of alignment at least partly explains why the ACE2 site was not found when mapping the Spike GP monomer structures. When looking at the individual FTMap results for the Spike GP open monomer structures, 15% did have at least a weak hot spot at the ACE2 binding site of the RBD domain.

The agreement of FTMove results with known binding sites is summarized in Table 2, and FTMove statistics are given in Tables S2–S10. For all ligandable sites, FTMove-site contacts to the protein overlap with key residues identified in the literature for the site or from the protein–ligand complex structure. The one exception is for the RdRp site that is adjacent to the catalytic triad, and in that case, an adjacent residue (L758) is identified as a contact to the FTMove site.

### 3.2. Ranking and Correlation Analysis

**Figure 5:**
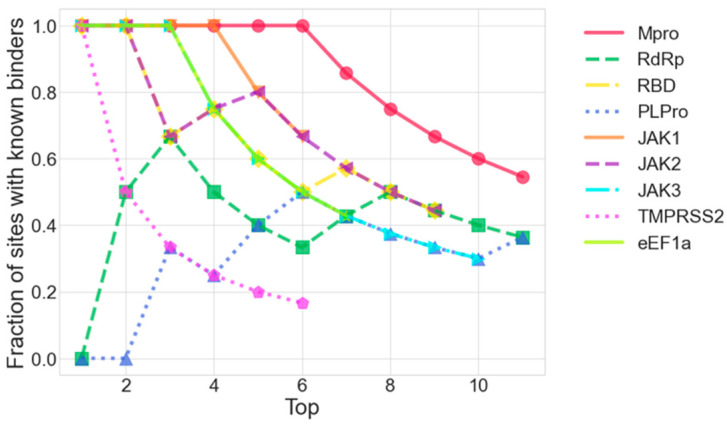
Recall in the Top X ranking by FTMove of sites with experimentally known binders for each target.

The enrichment, or “recall”, of the known binding sites for each target by the FTMove ranking is shown in Figure 5. For Mpro, a well-studied enzyme, the plot shows that the top ranked six sites are all known, ligandable sites. Similarly, for JAK1, the top-ranked four sites are all ligandable. For JAK3 and eEF1a, the top-ranked three sites are ligandable, while for JAK2 and Spike GP RBD, the top two are. For RdRp, the top-ranked site is not known to be ligandable, but the second and third are.

**Figure 6:**
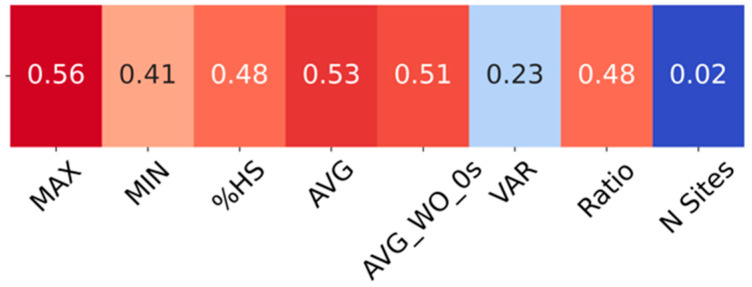
Correlation of each feature with manual ligandability label. The correlation scale is from red (1) to blue (0).

The correlation between each FTMove feature and the manual ligandability label was also calculated. The correlation coefficient varies from 0 to 1, and 1 indicates strong correlation. MAX, AVG of probe clusters, and AVG_WO_0s had correlation coefficients greater than 0.5, and %HS and Ratio had correlation coefficients above 0.45 (Figure 6). Ranking of FTMove sites by the sum of normalized MAX, AVG, AVG_WO_0s, %HS, and Ratio features did not improve the “recall” over the original FTMove ranking of the sum of MAX, AVG, AVG_WO_0s.

### 3.3. UMAP Results

**Figure 7:**
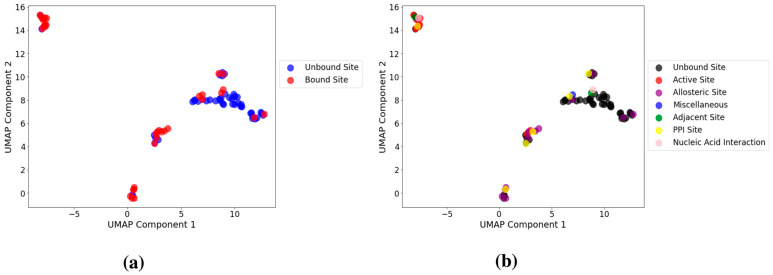
UMAP projection of FTMove site feature vectors. In (**a**) FTMove sites colored by their binary label of ligandable vs. not. In (**b**) binding sites colored by their category or specific label. Across the eight targets, a total of 81 FTMove sites are plotted.

A UMAP analysis was performed to reduce the dimensionality of the feature space and determine if the projection could separate sites with experimentally known binders from those without in a meaningful way. Each point in the UMAP projection plot (Figure 7) represents an FTMove site in one of the nine structures, and each site has eight features associated with it. In the UMAP projection, the sites form four clusters: three primarily with sites labeled as bound sites and one primarily with unbound sites. When the sites are colored based on their label/category, active sites (and adjacent to active sites) tend to cluster together on the bound site side of the plot, as do allosteric sites (Figure 7b).

**Figure 8:**
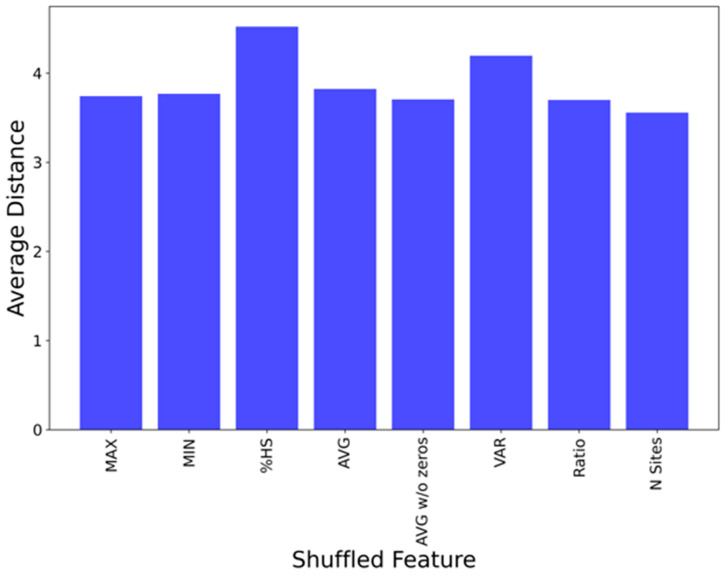
Sensitivity of UMAP components to each feature.

To tease out which features contribute most significantly to the UMAP components, a sensitivity analysis was done. This involved removing randomly shuffling one feature (out of the seven) at a time, regenerating the UMAP projection, and evaluating the average pairwise distance of each point (binding site) on the new projection vs. the original. Figure 8 shows that the UMAP components are most sensitive to %HS and VAR. The other features all contribute to a similar but lesser extent to the UMAP sensitivity.

### 3.4. Effect of Structure Resolution and Experimental Method on Mapping Results

**Figure 9:**
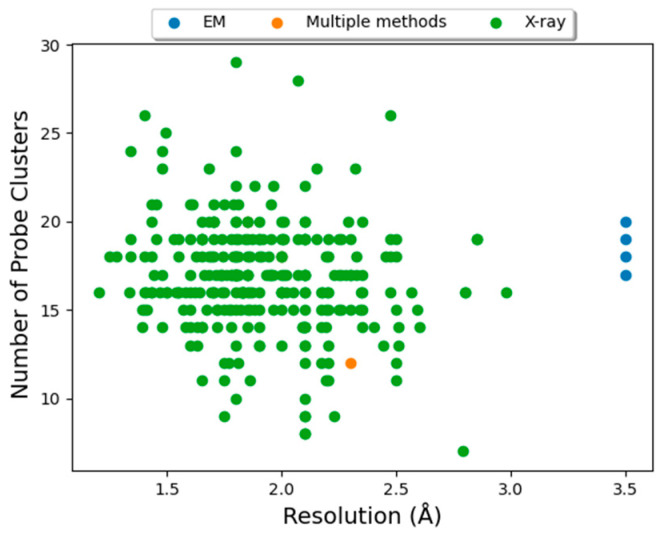
Effect of structure quality on hot spot score at the active site of Mpro. The number of probe clusters (MAX) in the FTMap hot spot corresponding to the active site of Mpro is plotted vs. structure resolution for each of the 293 structures of Mpro analyzed by FTMove. Points are colored by the method of structure determination.

Due to recent advances, cryogenic-electron microscopy (cryo-EM) is increasingly being used for protein structure determination. As such, a number of the more recent SARS-CoV-2 target structures have been determined using cryo-EM, typically at a somewhat lower resolution than those determined by X-ray crystallography. Mpro was taken as a case study, since its active site is well defined, and a large number of structures have been determined by different methods, to investigate the effect of structure resolution and method of determination on the quality of the mapping results. Figure 9 shows that at the active site hot spot, there is no correlation between the score (number of probe clusters where higher is better) and the structure resolution (Figure S1 shows the same plots for the other hot spots). Furthermore, structures solved by Cryo-EM, while exhibiting lower resolution (3.5 vs. typically less than 2.5 Å) yielded typical mapping scores for the hot spot at the active site (all four of which were greater than 16).

## 4. Discussion

FTMove was used to identify and rank binding sites for a set of nine COVID-19 drug/potential drug targets. A systematic review of the known ligandable sites for the same set of nine targets was also carried out to provide a basis for comparison to the computational approach. A total of 29 experimentally known sites were found, all of which were also identified by FTMove. In addition, of those 29 sites, all had MAX of 16 or greater except for one PLPro site (05), which has a score of 15 and is a PPI, and two RdRp sites (06 and 07) which correspond to an RNA-binding site (MAX 11) and an adjacent to active site (MAX 13). PPI and adjacent to active may all be expected to correspond to weaker hot spots, and RNA-binding sites are typically highly charged. Active sites are typically ranked in the top three, with the exception of PLPro. The PLPro active site is ranked fifth (Site 04) but has a very high score overall (MAX 20).

FTMove identifies 19 additional binding sites, which are ranked by the FTMove score higher or equal to the last site in the ranked list with MAX ≥ 16. Two of these have MAX score < 16. The remaining 17 sites (over eight of the nine targets) may be false positives or may in fact be sites that could bind ligands. If sites with %HS < 4 are eliminated, that number drops to eight sites (over four targets, RdRp, Spike GP RBD, PLPro, and TMPRSS2).

Compared to its parent server, FTMap, FTMove has the ability to map multiple structures simultaneously, which allows for the simultaneous identification of allosteric and potentially cryptic sites that are not present in every structure, in addition to active sites. Here we showed that FTMove can be employed to rapidly, and in an automated fashion, identify ligandable sites across all known/suspected targets for a given disease indication. This capability is particularly valuable for any swiftly emerging infectious disease threat. In fact, for future pandemics due to known pathogens of concern, binding sites can be pre-computed and stored in a database to focus research efforts on therapeutics design on the most promising targets. Our previous work has shown that AF2 models are sufficient for hot spot mapping, and as such, the existence of experimental structures, while desirable, is not required [59]. Additionally, at least for the Mpro active site (Site 00), we did not see a correlation between the number of probes in the hot spot consensus site (MAX) and the resolution of the structure; this result suggests cryo-EM structures (with resolution ≤ 3.5 Å) are also sufficient for hot spot mapping.

While the number of structures available has a significant impact on the overall FTMove results, meaningful results can be obtained even with a few structures. If only a few structures are available, which is often the case for applications to novel targets, the available structures can be augmented by high-confidence ColabFold models, as was done here for eEF1a, or snapshots from stable regions of molecular dynamics trajectories. For eEF1a, using only the five X-ray structures, two of the three ligandable sites were identified; augmenting the structures with the ColabFold models enables the identification of the third site.

Taking the experimentally known ligand-bound sites as the ground truth, a UMAP dimensionality reduction analysis of the FTMove features for each identified binding site was able to separate sites with bound ligands from those that are unbound. In this analysis, up to the top 11 sites identified by FTMove for each target regardless of the score were considered (as given in Tables S2–S10). Three clusters of largely bound sites were on one side of the plot, and one cluster of largely unbound sites was on the other side of the plot. One of the three bound site clusters corresponds to active sites (and adjacent to active) and the other two primarily to allosteric sites. This result further affirms the utility of FTMove to rapidly identify actionable sites across a range of targets for a given indication.

A limitation of the approach may be in assessing large proteins with multiple domains (such as Spike GP, RdRp, and PLPro) and in particular those that undergo large-scale conformational changes. As we saw with Spike GP, it may be necessary to perform the FTMove calculation on a single domain to obtain fully accurate results. When this is done, care needs to be taken to eliminate false sites that are at the interfaces with the missing domains. In future implementations, the FTMove server may be enhanced to streamline the separation of distinct conformations of structures due to large-scale movements and to eliminate automatically sites at domain interfaces when the structure has been trimmed to one domain. In addition, there could be sites on the surface of the multimeric proteins that would also not be detected due to the current computational limits, although, at least in the case of the Spike GP, all known, ligandable sites were identified using the monomer.

Attempts to refine the FTMove scoring function that ranks the sites for a given target, by considering different combinations of FTMove features, did not improve the “recall”. While MAX and AVG correlate most closely with the manual ligandability label, an analysis of the sensitivity of the UMAP components to FTMove features indicated that %HS and VAR contributed more significantly than the others. These data suggest that with a larger training set, it may be possible to develop a machine learning model capable of more accurately ranking the sites.

In summation, the FTMove method proved to be an efficient tool for binding site identification, as it was able to locate the known, ligandable binding sites based on the comprehensive literature review of COVID-19 drug targets. The approach, together with recent advances in AI-based structure prediction, has the potential in the future to transform our readiness for developing medical countermeasures for emerging viral and other diseases through structure-guided methods. Future enhancements may further enable the selection of the most promising druggable sites.

## Figures and Tables

**Table 1:** SARS-CoV-2 therapeutic targets selected for analysis.

Protein Target	UniProt ID	Organism Structure	PDB ID for FTMove ^a^	Structures in PDB ^b^	FDA Approved Drugs/Vaccines
Mpro (nsp5)	P0DTD1	Viral	7S82	1458	Paxlovid
RdRp (nsp12)	P0DTD1	Viral	7ED5	65	Veklury, Lagevrio ^c^
Spike Glycoprotein	P0DTC	Viral	6VXX	1709	Comirnaty, Spikevax, Novavax ^c^
PLPro (nsp3)	P0DTD1	Viral	6WX4	62	-
JAK1	P23458	Human	4EHZ	45	-
JAK2	O60674	Human	2XA4	104	-
JAK3	P52333	Human	5TTV	40	-
TMPRSS2	O15393	Human	-	7	-
eEF1a	Q05639	Human	-	8 (5) ^d^	-

^a^ PDB ID used as FTMove input. Chain A used for all. ^b^ Based on a sequence identity search with a 90% cutoff. ^c^ Emergency Use Authorization. ^d^ Only 5 useable; supplemented with ColabFold models.

**Table 2:** Summary of overlap of known ligands with FTMove sites.

Target Name	Binding Site Description	FTMove Site	Key Residues ^a^	FTMove Site Contacts ^b^	Site Location	Ref. PDB ^c^	Ref.
Mpro	Active Site (Nirmatrelvir)	00/02	**H41**, **C145**, **H163**	**H41**, C44, T45, D48-L50, P52, Y54, L141-**C145, H163**-L167, H172, V186-T190, Q192	Figure 2a left image, Green/Red	6Y2F	[48]
Mpro	Allosteric Site 1	01/03	**Q107-Q110**, **N151**, **I200**, **V202-N203**, **H246**, **I249**, **T292-F294**, **R298**	**Q107-Q110**, **N151-D153**, **I200-N203**, **E240**, **H246, I249**, **T292**-D295, **R298**, F305	Figure 2a, right image, Purple	7AGA	[33]
Mpro	Allosteric Site 2	04	**M6-F8**, **Q127**, **D295**, **R298**-**Q299**, S301-G302	F3, R4, **M6-F8**, T111, **Q127**, L282, F291, **D295**, V296, **R298**, **Q299**, F305	Figure 2a, left image, Purple	5RFA	[33]
Mpro	Surface Pocket (AR-42)	05	**L272**, G275, M276, L286, **L287**	R131, T199, Y239, L271, **L272**, **L287**, D289	Figure 2a, left image, Blue	7AXO	[49]
RdRp	Nsp8 Binding Site	01	**F326**, **P328**, V330, R331, S384	P323, T324, S325, **F326**, G327, **P328**, Y346, F396, V398, S664, M666, V675	Figure 2b, left image, Yellow	6M71	n/a
RdRp	G-Pocket	02	V31, **R33**, **F35**, C53, **R55**, Y69, V71, **K73**, **R116**, **T120**, **K121**, **T123**, **D208, N209**, **Y217**, **D218**, F219, G220, D221	**R33**-N39, K50, **R55**, **K73**, **R116**, L119, **T120-T123**, D126, T206, **D208**, **N209**, D211, **Y217**, **D218**, Y728, R733	Figure 2b, right image, Red	8GWE	[37]
RdRp	RNA-Binding Site	06	**S501**, K545, **R569**, S682-A685, Y689	**S501**, G503, Y516, G559, V560, I562, T565, **R569**, **G683**, **D684**	Figure 2b, left image, Pink	7B3B	[50]
RdRp	Adjacent to Catalytic Triad/ Remdesivir Site	07	S759, D760, D761, D618	V588, G590, T591, W598, L758, F812, C813	Figure 2b, left image, Green	7B3B	[50]
Spike GP	Heme Binding Site	00	**W104**, **V126**, I129, **F192**, **F194**, **I203**, **L226**	S94, N99, I101-**W104**, L117, I119, N121, **V126**, I128, **F192**, **F194**, F201, **I203**, Y204, H207, **L226**, V227, L229	Figure 3a/b, left image, Purple	7NT9	[46]
Spike GP	Free Fatty Acid Binding Site	04	**F338**, A363, **Y365**, **Y369**, **F377**, **V395**, **F515**	P337, **F338**, I358, **Y365**, L368, **Y369**, **F377**, C379, P384, L387, **V395**, C432, I434, L513, **F515**, V524	Figure 3a, right image. Purple	7E7B	[47]
Spike GP	NTD-directed Antibody Binding Site	07/08	**E156**, R246, Y248, D253,	W64, H66, A67, T95-S98, I100, L118, V120, N122, V127, F133-C136, P139-L141, V143, **E156**-S162, V213-D215, L241, A243-H245, A264	Figure 3b, left image, Yellow	7L2F	[51]
Spike GP RBD	Heme Binding Site	01	**F338**, **A363**, **Y365**, Y369, F377, V395, F515	L335, C336, **F338**, F342-A344, V362-V367, L368, S371, S373, F374, W436, N440, L441, R509	Figure 3c, left image, Purple	7E7B	[47]
Spike GP RBD	ACE2 Binding Site	03	S438–E506	R403, E406, Q409, K417, I418, **Y453**, **S494-Q498**, **N501**, **Y505**	Figure 3c, left image, Yellow	7M17	[52]
PLPro	Ubiquitin Binding Site	02	**R166**, E167, Q174, **E203**, **M208**	**R166**, R183, L199, V202, E203, M206, Y207, **M208**	Figure 2c, Yellow	6XAA	[53]
PLPro	Catalytic Site	04	**C111**, H272, D286	N109, **C111**, Y112, G163, D164, R166, M208, S245, A246, Y264, Y268, Q269, G271, Y273, T301, D302	Figure 2c, Red	7LBS	[54]
PLPro	ISG15 Interaction Site	05	**Y171**, **Q174**	T74, D76, F79, R82, Y154, N156, **Y171**, **Q174**, H175	Figure 2c, Yellow	6YVA	[38]
PLPro	Allosteric Site	10	**V57**, T74, D76	**V57**, P59, L80	Purple	7OFS	[38]
JAK1	Active Site (ATP)	00	**L881**, **E883**, **V889**, A906, **M956**, **E957**, **F958, L959**, G962, **S963**, **E966**, **R1007**, **N1008**, **L1010**, **G1020**, **D1021**	**L881**-G887, **V889**, **M956**-**L959**, **S963**, K965, **E966**, **R1007**, **N1008**, **L1010**, **G1020**, **D1021**	Figure 2d, Red	4IVD	[55,56]
JAK1	MT3 Allosteric Site	01	**K908**, **E925**, L929, V938, M956, **D1003**, D1021-L1024	G884-K888, **K908**, S909, H918, D921, L922, **E925**, **D1003**, R1007, **G1023**	Figure 2d, Purple	4AN2	[55]
JAK1	SOCS1 Binding Site	02	**D1040-S1043**	**D1042, S1043**, F1046	Figure 2c, Yellow	6C7Y	[57,58]
JAK1	PDIG Allosteric Site	03	P969, **E1073**, **C1078**-S1080, S1082-P1084	R1007, **E1073**, **C1078**, **D1079**	Figure 2d, Purple	3JVS	[55]
JAK2	Active Site (ATP)	00	**L855**, **G856**, **E930**-**L932**	**L855**-K857, V863, K882, V911, M929-**L932**, G935, D976, R980-L983, G993-F995	Figure 2e, Red	4C61	[55]
JAK2	MT3 Allosteric Site	01	**K882**, **E898**, L902, V911, **D976**, D994-**L997**	G858-G861, **K882**, L884, H886, H891, **E898**, **D976**, R980, **G996**, **L997**, E1012, P1017	Figure 2e, Purple	4AN2	[55]
JAK2	DFG-out Site	03/04	V863, A880, I901, L927, M929, L932, **I973**, **G993**-**F995, L997-K999**	E898, **I973**, H974, I992-D994, **F995-K999**	Figure 2e, Purple	2W1I	[55]
JAK3	Active Site (ATP)	00	**M902**, **L905**, **C909**, **N954**, **D967**	L828, V836, A853, K855, E871, **M902**, E903, **L905**, G908, **C909**, R953-**D967**	Figure 2f, Red	4QPS	[55]
JAK3	MT3 Allosteric Site	01	**N832**, **K855**, **E871**, L875, V884, M902, **D949**, D967-**L970**, S989	G831-G834, **K855**, L857, D867, **E871**, R948, **D949**, **G969**, **L970**	Figure 2f, Purple	4AN2	[55]
JAK3	PDIG Allosteric Site	02	**R911**, Q915, R916, **C1024**-K1026, S1029	**R911**, R953, W993, E1019, **C1024**, P1030	Figure 2f, Purple	3JVS	[55]
TMPRSS2	Catalytic Triad	00	H296, D345, **S441**	S436, C437, Q438, **S441**, T459, S460, W461, G464, V473, Y474	Figure 2g, Red	7MEQ	[40]
eEF1a	Translation Inhibition Site	00	**Y141**, **Q343**, R382, A399, **R423**, V437	L138, **Y141**, T142, I181, **Q343**, **R423**, F424, V435, G436	Figure 2h, right image, Red	5LZS	[45]
eEF1a	aa-tRNA Binding Site	01	T6-I8, Y85-V87, **E293**, H295, H296	V262, V264, **E293**, N307, V308, G309	Figure 2h, right image, Pink	5LZS	[42,45]
eEF1a	GDP Binding Site	02	D17, **K20-T22**, D156, S194-W196	**K20-T22**, D61, T72, G94	Figure 2h, right image, Red	5LZS	[43]

^a^ Residues given for the site in the corresponding reference, or, if none described, those within 3 Å of the ligand in the reference PDB. ^b^ Residues within 3 Å of the probes in the FTMove site mesh; those residues that are in agreement with Key Contacts are in bold. ^c^ PDB ID of a structure that has the corresponding small molecule, protein, nucleic acid in the site.

## Data Availability

All PDB codes for the structures and ColabFold models studied in this work are provided within the published article and its Supporting Information. PyMOL sessions with FTMap sites identified in this study can be found in the following repository: https://github.com/marialzs/ftmove_sars_cov_2 (accessed on 25 August 2024). The FTMove and FTMap servers are available to use free of charge for academic and governmental purposes at https://ftmap.bu.edu (accessed on 25 August 2024).

## Supplementary Materials

The following supporting information can be downloaded at: https://www.mdpi.com/article/10.3390/v16111647/s1, Table S1: Spike GP structures utilized in FTMove analysis with conformational state indicated; Tables S2–S10: Tables of FTMove site statistics by target; Table S10: Spike GP structures utilized in FTMove analysis with conformational state indicated; Figure S1: Number of probe clusters per binding site in Mpro versus structure resolution; a CVS file with the seeds used with Colabfold.
