# Supplementary material for: Identification and Ranking of Binding Sites from Structural Ensembles: Application to SARS-CoV-2
Source: Viruses. 2024 Oct 22;16(11):1647. doi: 10.3390/v16111647 (PMC11599001; doi:10.3390/v16111647)
Supplement: Supplementary file 1 [file viruses-16-01647-s001.zip › SupplementaryInfo_v6.pdf]

Supplementary Information

Identification and Ranking of Binding Sites from Structural Ensembles: Application to SARS-CoV-2

Maria Lazou [1], Ayse A. Bekar-Cesaretli [2], Sandor Vajda [1,2] , and Diane Joseph-McCarthy [1,2,*]

1 Department of Biomedical Engineering, Boston University
2 Department of Chemistry, Boston University
* Correspondence: djosephm@bu.edu

Supplementary information
This file includes:

Table S1. Spike GP structures utilized in FTMove analysis with conformational state indicated

Table S2 – Table S10. Tables of FTMove site statistics by target

Figure S1. Number of probe clusters per binding site in Mpro versus structure resolution

Table S1. Spike GP Structures Utilized in FTMove Analysis with Conformational State Indicated

PDB ID	State	PDB ID	State	PDB ID	State	PDB ID	State	PDB ID	State
6XCM.B	Open	7DK4.B	Open	6WPS.B	Closed	6ZOZ.C	Closed	7DK7.C	Closed
6XCM.C	Open	7DK5.A	Open	6WPS.E	Closed	6ZP0.A	Closed	7JJI.A	Closed
6XCN.A	Open	7DK6.A	Open	6WPT.A	Closed	6ZP0.B	Closed	7JJI.B	Closed
6XCN.C	Open	7DK6.B	Open	6WPT.C	Closed	6ZP0.C	Closed	7JJI.C	Closed
6XCN.E	Open	7DK7.A	Open	6X6P.C	Closed	6ZP1.A	Closed	7JV4.A	Closed
6XM0.B	Open	7DK7.B	Open	6XEY.A	Closed	6ZP1.B	Closed	7JV4.C	Closed
6XM3.B	Open	7JVC.A	Open	6XEY.B	Closed	6ZP1.C	Closed	7JV6.A	Closed
6XM4.B	Open	7JVC.B	Open	6XEY.C	Closed	6ZP2.A	Closed	7JV6.B	Closed
6Z43.A	Open	7JVC.E	Open	6XF5.A	Closed	6ZP2.B	Closed	7JV6.E	Closed
6Z97.B	Open	7JWB.B	Open	6XF5.B	Closed	6ZP2.C	Closed	7JWB.A	Closed
6ZDH.A	Open	7K4N.A	Open	6XF5.C	Closed	6ZP5.B	Closed	7JWY.A	Closed
6ZDH.B	Open	7K4N.B	Open	6XF6.A	Closed	6ZP7.C	Closed	7JWY.B	Closed
6ZDH.C	Open	7K4N.E	Open	6XF6.C	Closed	6ZXN.B	Closed	7JWY.C	Closed
6ZGG.B	Open	7K8T.B	Open	6XM0.A	Closed	6ZXN.C	Closed	7JZL.A	Closed
6ZHD.A	Open	7K8U.B	Open	6XM3.A	Closed	7A29.C	Closed	7K43.A	Closed
6ZP5.A	Open	7K8U.C	Open	6XM3.C	Closed	7A4N.A	Closed	7K43.B	Closed
6ZP7.A	Open	7K8X.C	Open	6XM4.A	Closed	7A4N.B	Closed	7K43.E	Closed
6ZXN.A	Open	7K8Y.D	Open	6XM4.C	Closed	7A4N.C	Closed	7K8S.A	Closed
7A29.A	Open	7K8Y.E	Open	6XM5.A	Closed	7A93.C	Closed	7K8S.B	Closed
7A29.B	Open	7KJ2.B	Open	6XM5.B	Closed	7A94.B	Closed	7K8S.C	Closed
7A93.A	Open	7KJ3.A	Open	6XM5.C	Closed	7A94.C	Closed	7K8T.A	Closed
7A93.B	Open	7KJ3.B	Open	6Z43.B	Closed	7AD1.A	Closed	7K8T.C	Closed
7A94.A	Open	7KJ4.A	Open	6Z43.C	Closed	7AD1.C	Closed	7K8U.A	Closed
7A95.A	Open	7KJ4.B	Open	6Z97.A	Closed	7BYR.B	Closed	7K8W.A	Closed
7A95.C	Open	7KJ4.C	Open	6Z97.C	Closed	7CAI.C	Closed	7K8W.G	Closed
7A96.A	Open	7KJ5.B	Open	6ZB4.A	Closed	7CHH.B	Closed	7K8X.A	Closed
7CAI.A	Open	7KL9.A	Open	6ZB4.B	Closed	7CHH.C	Closed	7K8X.B	Closed
7CHH.A	Open	7KL9.C	Open	6ZB4.C	Closed	7CWL.A	Closed	7K8Y.B	Closed
7CT5.A	Open	7KMS.A	Open	6ZB5.A	Closed	7CWM.<br>A	Closed	7K8Z.A	Closed
7CT5.B	Open	7KMS.B	Open	6ZB5.B	Closed	7CWM.<br>C	Closed	7K8Z.C	Closed
7CT5.C	Open	7KMS.C	Open	6ZB5.C	Closed	7DCX.D	Closed	7K90.A	Closed
7CWL.C	Open	7KMZ.A	Open	6ZGE.A	Closed	7DD2.D	Closed	7K90.B	Closed
7CWM.<br>B	Open	7KMZ.B	Open	6ZGE.B	Closed	7DD8.D	Closed	7K90.C	Closed
7CWN.<br>A	Open	7KNB.B	Open	6ZGE.C	Closed	7DD8.E	Closed	7KDG.A	Closed

7CWN.B	Open	7KNE.B	Open	6ZGG.A	Closed	7DDD.A	Closed	7KDG.B	Closed
7CWN.C	Open	7KNH.A	Open	6ZGG.C	Closed	7DDD.B	Closed	7KDG.C	Closed
7CWU.B	Open	7KNH.B	Open	6ZGH.B	Closed	7DDD.C	Closed	7KDH.A	Closed
7CWU.C	Open	7KNI.A	Open	6ZGH.C	Closed	7DDN.A	Closed	7KDH.C	Closed
7DCC.E	Open	7KNI.B	Open	6ZGI.A	Closed	7DDN.B	Closed	7KJ2.A	Closed
7DCC.I	Open	7KNI.C	Open	6ZHD.B	Closed	7DF3.A	Closed	7KJ2.C	Closed
7DCC.K	Open	7KSG.A	Open	6ZHD.C	Closed	7DF3.B	Closed	7KJ3.C	Closed
7DCX.C	Open	7KSG.B	Open	6ZOW.C	Closed	7DF3.C	Closed	7KJ5.A	Closed
7DCX.K	Open	7KSG.C	Open	6ZOX.A	Closed	7DF4.C	Closed	7KJ5.C	Closed
7DD2.C	Open	6VSB.C	Closed	6ZOX.B	Closed	7DF4.D	Closed	7KKK.A	Closed
7DD2.K	Open	6VXX.A	Closed	6ZOX.C	Closed	7DK3.A	Closed	7KKK.C	Closed
7DD8.C	Open	6VXX.B	Closed	6ZOY.A	Closed	7DK3.B	Closed	7KKK.E	Closed
7DDN.C	Open	6VXX.C	Closed	6ZOY.B	Closed	7DK4.C	Closed	7KKL.A	Closed
7DF4.B	Open	6VYB.A	Closed	6ZOY.C	Closed	7DK5.B	Closed	7KKL.C	Closed
7DK4.A	Open	6VYB.C	Closed	6ZOZ.A	Closed	7DK5.C	Closed	7KKL.D	Closed
7DK4.B	Open	6WPS.A	Closed	6ZOZ.B	Closed	7DK6.C	Closed	7KMZ.C	Closed
7KNH.C	Closed	7KNE.C	Closed	7KNE.A	Closed	7KNB.C	Closed	7KNB.A	Closed

Table S2. FTMove Site Statistics for Mpro [a]

Target	*Site Number*										
MPro	***0***	***1***	***2***	***3***	***4***	***5***	***6***	***7***	***8***	***9***	***10***
**MAX**	29.0	27.0	25.0	20.0	28.0	19.0	13.0	16.0	16.0	9.0	9.0
**MIN**	7.0	7.0	8.0	0.0	0.0	0.0	0.0	0.0	0.0	0.0	0.0
**%HS**	70.3	66.9	59.7	15.0	6.1	2.4	0.0	0.3	0.3	0.0	0.0
**AVG**	16.8	16.9	16.8	10.5	5.1	6.5	3.8	0.4	0.3	0.1	0.3
**AVG_WO_0s**	16.8	16.9	16.8	11.2	6.0	6.9	5.3	2.9	2.3	4.3	1.9
**VAR**	10.1	12.8	16.3	27.4	28.3	13.5	11.5	1.9	1.3	0.8	1.0
Ratio	*1*	*0.95*	*0.85*	*0.21*	*0.09*	*0.03*	*0*	*0.01*	*0.01*	*0*	*0*
Total Sites	***11***	***11***	***11***	***11***	***11***	***11***	***11***	***11***	***11***	***11***	***11***

[a] Font colors correspond to the site label: red for active sites, purple for allosteric sites, green for adjacent (to active) sites, yellow for protein-protein interaction sites, pink for nucleic acid binding sites, and blue for miscellaneous sites

Table S3. FTMove Site Statistics for RdRp [a]

Target	*Site Number*										
RdRp	***0***	***1***	***2***	***3***	***4***	***5***	***6***	***7***	***8***	***9***	***10***
**MAX**	25.0	17.0	16.0	14.0	19.0	21.0	11.0	13.0	15.0	11.0	12.0
**MIN**	8.0	2.0	0.0	5.0	0.0	0.0	0.0	0.0	0.0	0.0	0.0
**%HS**	33.3	3.5	12.3	0.0	1.8	1.8	0.0	0.0	0.0	0.0	0.0
**AVG**	14.2	9.0	8.1	9.3	2.4	1.3	5.6	2.5	0.8	3.6	2.4
**AVG_WO_0s**	14.2	9.0	10.8	9.3	6.3	3.7	7.0	5.1	4.6	4.5	4.1
**VAR**	14.0	8.1	32.2	5.0	17.0	11.0	13.1	13.4	6.2	12.1	7.0
Ratio	*1*	*0.10*	*0.37*	*0*	*0.05*	*0.05*	*0*	*0*	*0*	*0*	*0*
Total Sites	***16***	***16***	***16***	***16***	***16***	***16***	***16***	***16***	***16***	***16***	***16***

[a] Font colors correspond to the site label: red for active sites, purple for allosteric sites, green for adjacent (to active) sites, yellow for protein-protein interaction sites, pink for nucleic acid binding sites, and blue for miscellaneous sites

Table S4. FTMove Site Statistics for Spike GP [a]

Target	*Site Number*										
Spike GP RBD	***0***	***1***	***2***	***3***	***4***	***5***	***6***	***7***	***8***	***9***	***10***
**MAX**	21.0	25.0	24.0	22.0	22.0	18.0	22.0	15.0	9.0		
**MIN**	0.0	4.0	0.0	0.0	0.0	0.0	0.0	0.0	0.0		
**%HS**	76.2	40.5	28.6	42.9	14.3	16.7	7.1	0.0	0.0		
**AVG**	16.2	14.8	10.3	10.3	9.2	8.9	2.1	1.4	0.7		
**AVG_WO_0s**	17.5	14.8	12.7	13.1	10.5	10.7	9.9	3.6	3.8		
**VAR**	32.8	15.9	70.6	61.4	37.5	33.9	29.7	7.0	3.2		
Ratio	1	0.5	0.4	0.6	0.2	0.2	0.1	0	0		
Total Sites	9	9	9	9	9	9	9	9	9		
Spike GP	***0***	***1***	***2***	***3***	***4***	***5***	***6***	***7***	***8***	***9***	***10***
**MAX**	28	25	29	25	27	18	25	21	23	17	14
**MIN**	0	0	0	0	0	0	0	0	0	0	0
**%HS**	41.99	35.23	21.00	12.46	3.56	0.36	1.07	3.20	0.36	1.07	0.00
**AVG**	13.49	13.22	6.55	9.36	2.80	0.06	1.01	3.93	0.68	6.60	0.14
**AVG_WO_0s**	14.75	13.41	15.59	10.27	8.04	18.00	7.89	8.69	9.05	7.51	12.67
**VAR**	40.39	20.91	80.85	31.93	27.17	1.15	10.97	28.51	7.69	17.32	1.71
Ratio	1.00	0.84	0.50	0.30	0.08	0.01	0.03	0.08	0.01	0.03	0.00
Total Sites	40	40	40	40	40	40	40	40	40	40	40
Spike GP Open [b]	***0***	***1***	***2***	***3***	***4***	***5***	***6***	***7***	***8***	***9***	***10***
**MAX**	28.00	25.00	25.00	21.00	23.00	21.00	18.00	22.00	18.00	18.00	13.00
**MIN**	0.00	0.00	0.00	0.00	0.00	0.00	0.00	0.00	0.00	0.00	0.00
**%HS**	46.74	35.87	7.61	1.09	2.17	5.43	4.35	1.09	2.17	1.09	0.00
**AVG**	14.22	13.80	8.51	0.23	1.25	4.54	2.95	1.24	1.00	0.98	0.14
**AVG_WO_0s**	15.21	14.11	10.04	21.00	11.50	8.89	10.04	6.00	9.20	7.50	13.00
**VAR**	40.00	23.54	30.58	4.79	17.00	31.15	27.37	11.35	12.04	9.45	1.84
Ratio	1.00	0.77	0.16	0.02	0.05	0.12	0.09	0.02	0.05	0.02	0.00
Total Sites	33	33	33	33	33	33	33	33	33	33.00	33.00
Spike GP Closed [b]	***0***	***1***	***2***	***3***	***4***	***5***	***6***	***7***	***8***	***9***	***10***
**MAX**	30.00	27.00	33.00	25.00	26.00	24.00	18.00	16.00	19.00	17.00	19.00
**MIN**	0.00	0.00	0.00	0.00	0.00	0.00	0.00	0.00	0.00	0.00	0.00
**%HS**	34.57	37.04	9.26	33.33	3.70	1.85	2.47	0.62	3.09	0.62	0.62
**AVG**	10.17	12.96	8.63	12.80	2.80	0.98	3.98	6.48	3.10	0.12	1.67
**AVG_WO_0s**	16.32	13.99	9.99	12.88	10.30	9.29	8.96	7.29	5.12	10.00	5.65
**VAR**	89.75	34.66	38.26	19.33	31.09	13.38	27.17	17.68	15.24	1.84	10.62
Ratio	1.00	1.07	0.27	0.96	0.11	0.05	0.07	0.02	0.09	0.02	0.02
Total Sites	36	36	36	36	36	36	36	36	36	36	36

[a] Font colors correspond to the site label: red for active sites, purple for allosteric sites, green for adjacent (to active) sites, yellow for protein-protein interaction sites, pink for nucleic acid binding sites, and blue for miscellaneous sites.

[b] Site 10 (a relatively weak site) on both the Spike GP Open and Closed monomer is at the monomer-monomer interface, and thus would not be a site in the trimer.

Table S5. FTMove Site Statistics for PLPro [a]

Target	*Site Number*										
PLPro	***0***	***1***	***2***	***3***	***4***	***5***	***6***	***7***	***8***	***9***	***10***
**MAX**	24.0	25.0	24.0	24.0	20.0	15.0	17.0	10.0	10.0	9.0	9.0
**MIN**	6.0	3.0	2.0	0.0	0.0	0.0	0.0	0.0	0.0	0.0	0.0
**%HS**	54.7	34.7	20.0	10.5	13.7	0.0	1.1	0.0	0.0	0.0	0.0
**AVG**	15.7	14.1	12.4	5.5	8.8	6.5	1.2	0.1	0.1	1.3	0.8
**AVG_WO_0s**	15.7	14.1	12.4	10.3	8.9	7.2	6.9	10.0	10.0	3.5	3.6
**VAR**	17.6	20.7	13.9	48.7	25.1	14.9	10.0	1.1	1.1	4.3	3.2
Ratio	*1*	*0.63*	*0.37*	*0.19*	*0.25*	*0*	*0.02*	*0*	*0*	*0*	*0*
Total Sites	***11***	***11***	***11***	***11***	***11***	***11***	***11***	***11***	***11***	***11***	***11***

[a] Font colors correspond to the site label: red for active sites, purple for allosteric sites, green for adjacent (to active) sites, yellow for protein-protein interaction sites, pink for nucleic acid binding sites, and blue for miscellaneous sites

Table S6. FTMove Site Statistics for JAK1 [a]

Target	*Site Number*										
JAK1	***0***	***1***	***2***	***3***	***4***	***5***	***6***	***7***	***8***	***9***	***10***
**MAX**	32.0	20.0	16.0	21.0	14.0	15.0					
**MIN**	14.0	3.0	3.0	0.0	0.0	0.0					
**%HS**	87.2	17.9	1.3	1.3	0.0	0.0					
**AVG**	19.1	11.4	8.7	1.9	3.5	2.2					
**AVG_WO_0s**	19.1	11.4	8.7	6.3	5.0	4.3					
**VAR**	12.9	18.0	4.8	15.6	9.8	10.3					
Ratio	*1*	*0.21*	*0.01*	*0.01*	*0*	*0*					
Total Sites	***6***	***6***	***6***	***6***	***6***	***6***					

[a] Font colors correspond to the site label: red for active sites, purple for allosteric sites, green for adjacent (to active) sites, yellow for protein-protein interaction sites, pink for nucleic acid binding sites, and blue for miscellaneous sites

Table S7. FTMove Site Statistics for JAK2 [a]

Target	*Site Number*									
JAK2	***0***	***1***	***2***	***3***	***4***	***5***	***6***	***7***	***8***	***9***	***10***
MAX	32.0	25.0	16.0	17.0	21.0	19.0	13.0	12.0	11.0		
MIN	8.0	4.0	0.0	0.0	0.0	0.0	0.0	0.0	0.0		
%HS	86.2	33.9	0.9	0.9	2.8	0.9	0.0	0.0	0.0		
AVG	18.9	14.1	9.3	0.2	2.8	4.6	3.0	2.3	2.3		
AVG_WO_0s	18.9	14.1	9.4	17.0	8.3	5.1	5.2	4.6	4.4		
VAR	14.8	19.1	6.5	2.7	25.2	15.1	11.1	8.6	7.4		
Ratio	1.00	0.39	0.01	0.01	0.03	0.01	0.00	0.00	0.00		
Total Sites	***10***	***10***	***10***	***10***	***10***	***10***	***10***	***10***	***10***		

[a] Font colors correspond to the site label: red for active sites, purple for allosteric sites, green for adjacent (to active) sites, yellow for protein-protein interaction sites, pink for nucleic acid binding sites, and blue for miscellaneous sites

Table S8. FTMove Site Statistics for JAK3 [a]

Target	*Site Number*										
JAK3	***0***	***1***	***2***	***3***	***4***	***5***	***6***	***7***	***8***	***9***	***10***
**MAX**	34.0	21.0	23.0	17.0	14.0	11.0	13.0	10.0	12.0	10.0	
**MIN**	14.0	6.0	0.0	0.0	0.0	0.0	0.0	0.0	0.0	0.0	
**%HS**	79.5	23.1	10.3	2.6	0.0	0.0	0.0	0.0	0.0	0.0	
**AVG**	19.8	12.1	6.8	3.7	2.1	3.9	2.3	2.8	0.9	1.1	
**AVG_WO_0s**	19.8	12.1	9.4	6.3	3.6	4.6	3.1	5.1	4.3	4.1	
**VAR**	25.8	16.8	40.1	19.6	11.6	7.3	6.4	9.1	5.7	4.9	
Ratio	*1*	*0.29*	*0.13*	*0.03*	*0*	*0*	*0*	*0*	*0*	*0*	
Total Sites	***10***	***10***	***10***	***10***	***10***	***10***	***10***	***10***	***10***	***10***	

[a] Font colors correspond to the site label: red for active sites, purple for allosteric sites, green for adjacent (to active) sites, yellow for protein-protein interaction sites, pink for nucleic acid binding sites, and blue for miscellaneous sites

Table S9. FTMove Site Statistics for TMPRSS2 [a]

Target	*Site Number*										
TMPRSS2	***0***	***1***	***2***	***3***	***4***	***5***	***6***	***7***	***8***	***9***	***10***
**MAX**	26.0	24.0	17.0	15.0	15.0	15.0	9.0				
**MIN**	0.0	0.0	3.0	0.0	0.0	0.0	0.0				
**%HS**	75.0	83.3	33.3	0.0	0.0	0.0	0.0				
**AVG**	17.3	17.5	12.5	1.3	1.8	1.5	1.3				
**AVG_WO_0s**	18.9	19.1	12.5	15.0	11.0	9.0	3.5				
**VAR**	47.0	41.2	15.5	18.8	21.2	18.8	4.1				
Ratio	2.3	2.5	1.0	0.0	0.0	0.0	*0*				
Total Sites	*6*	*6*	*6*	*6*	*6*	*6*	*6*				

[a] Font colors correspond to the site label: red for active sites, purple for allosteric sites, green for adjacent (to active) sites, yellow for protein-protein interaction sites, pink for nucleic acid binding sites, and blue for miscellaneous sites

Table S10. FTMove Site Statistics for eEF1a [a]

Target	*Site Number*										
eEF1a	***0***	***1***	***2***	***3***	***4***	***5***	***6***	***7***	***8***	***9***	***10***
**MAX**	26.0	22.0	20.0	19.0	14.0	9.0	9.0				
**MIN**	10.0	14.0	0.0	6.0	0.0	0.0	0.0				
**%HS**	66.7	67.7	53.1	3.1	0.0	0.0	0.0				
**AVG**	17.5	16.2	15.2	12.6	11.3	0.1	0.8				
**AVG_WO_0s**	17.5	16.2	15.4	12.6	11.5	9.0	4.9				
**VAR**	14.8	2.0	7.5	2.5	5.1	0.8	4.2				
Ratio	1.0	1.0	0.8	0.0	0.0	0.0	0.0				
Total Sites	7	7	7	7	7	7	7				

[a] Font colors correspond to the site label: red for active sites, purple for allosteric sites, green for adjacent (to active) sites, yellow for protein-protein interaction sites, pink for nucleic acid binding sites, and blue for miscellaneous sites

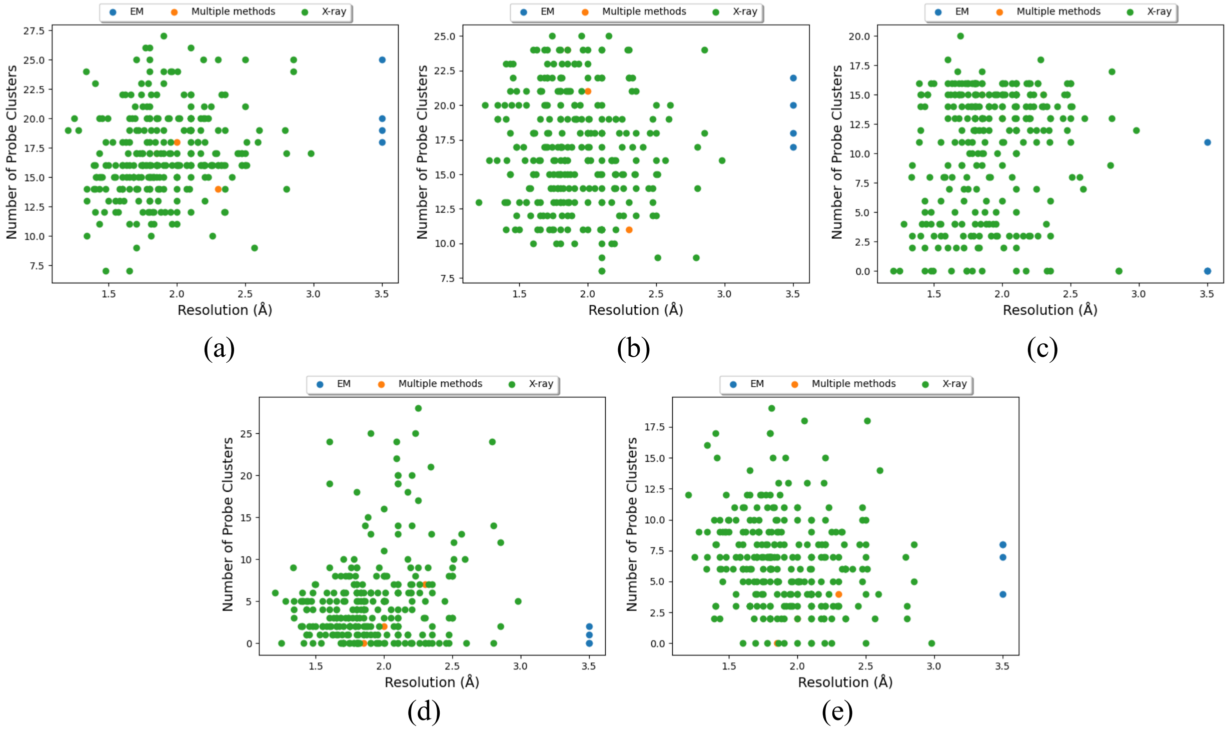

Figure S1. Number of probe clusters per binding site in Mpro versus structure resolution. In(a) for binding site 01 (b) binding site 02 (c) binding site 03 (d) binding site 04 (e) binding site 05.
